# Assessment of bacterial diversity in the cattle tick *Rhipicephalus *(*Boophilus*) *microplus *through tag-encoded pyrosequencing

**DOI:** 10.1186/1471-2180-11-6

**Published:** 2011-01-06

**Authors:** Renato Andreotti, Adalberto A Pérez de León, Scot E Dowd, Felix D Guerrero, Kylie G Bendele, Glen A Scoles

**Affiliations:** 1 EMBRAPA Beef Cattle, BR 262 km. 04, Caixa postal n. 154, Campo Grande, MS, 79.002-970, Brazil; 2 USDA-ARS Knipling-Bushland U.S. Livestock Insects Research Laboratory, 2700 Fredericksburg Rd., Kerrville, TX, 78028, USA; 3 Research and Testing Laboratory, Pathogenius, and Spirostat Technologies, 4321 Marsha Sharp Fwy., Lubbock, TX, 79407, USA; 4 USDA-ARS Animal Disease Research Unit, Washington State University, 3003 ADBF, Pullman, WA, 99164, USA

## Abstract

**Background:**

Ticks are regarded as the most relevant vectors of disease-causing pathogens in domestic and wild animals. The cattle tick, *Rhipicephalus *(*Boophilus*) *microplus*, hinders livestock production in tropical and subtropical parts of the world where it is endemic. Tick microbiomes remain largely unexplored. The objective of this study was to explore the *R. microplus *microbiome by applying the bacterial 16S tag-encoded FLX-titanium amplicon pyrosequencing (bTEFAP) technique to characterize its bacterial diversity. Pyrosequencing was performed on adult males and females, eggs, and gut and ovary tissues from adult females derived from samples of *R. microplus *collected during outbreaks in southern Texas.

**Results:**

Raw data from bTEFAP were screened and trimmed based upon quality scores and binned into individual sample collections. Bacteria identified to the species level include *Staphylococcus aureus, Staphylococcus chromogenes, Streptococcus dysgalactiae, Staphylococcus sciuri, Serratia marcescens, Corynebacterium glutamicum*, and *Finegoldia magna*. One hundred twenty-one bacterial genera were detected in all the life stages and tissues sampled. The total number of genera identified by tick sample comprised: 53 in adult males, 61 in adult females, 11 in gut tissue, 7 in ovarian tissue, and 54 in the eggs. Notable genera detected in the cattle tick include *Wolbachia*, *Coxiella*, and *Borrelia*. The molecular approach applied in this study allowed us to assess the relative abundance of the microbiota associated with *R. microplus*.

**Conclusions:**

This report represents the first survey of the bacteriome in the cattle tick using non-culture based molecular approaches. Comparisons of our results with previous bacterial surveys provide an indication of geographic variation in the assemblages of bacteria associated with *R. microplus*. Additional reports on the identification of new bacterial species maintained in nature by *R. microplus *that may be pathogenic to its vertebrate hosts are expected as our understanding of its microbiota expands. Increased awareness of the role *R. microplus *can play in the transmission of pathogenic bacteria will enhance our ability to mitigate its economic impact on animal agriculture globally. This recognition should be included as part of analyses to assess the risk for re-invasion of areas like the United States of America where *R. microplus *was eradicated.

## Background

Ticks are considered to be second only to mosquitoes as worldwide vectors of human diseases, but they are regarded as the most relevant vectors of disease-causing pathogens in domestic and wild animals [1]. The cattle tick, *Rhipicephalus *(*Boophilus*) *microplus*, hinders livestock production in tropical and subtropical parts of the world where it is endemic. For example, the economic impact on the cattle industry in Brazil by the cattle tick *R. microplus *is estimated to be two billion U.S. dollars annually [2]. In addition to direct economic loss associated with blood feeding by *R. microplus *during infestation, indirect effects are also significant due to the transmission of diseases like bovine babesiosis and anaplasmosis caused by the apicomplexan protozoans *Babesia bovis *and *Babesia bigemina*, and the bacterium *Anaplasma marginale*, respectively. The vector competency of *R. microplus *for *A. marginale *suggests that other microbial associations with this tick host may exist. However, quantitative and qualitative information on the composition of bacterial communities in *R. microplus *is scarce.

Seminal studies by Smith and Kilbourne at the end of the 19^th ^century demonstrating that *Rhipicephalus annulatus *transmitted *B. bigemina *triggered research on other microorganisms harbored by ticks [3,4]. Currently, our understanding of ticks as vectors of infectious agents has advanced to the point where some tick-borne bacterial diseases are considered an emerging infectious threat globally [5,6]. It is estimated that the number of described tick-borne pathogens affecting humans and animals will increase as research on tick biology and ecology progresses [7]. In some cases, species related to pathogenic bacteria were detected and identified in ticks before their effect on human health was fully determined [8]; but our knowledge of bacterial communities in ticks beyond pathogenic species is limited, even though the association between non-pathogenic bacteria and ticks was documented at the beginning of the 20^th ^century [9].

Bacteria are ubiquitous microorganisms and some have evolved symbioses with ticks. In addition to transmitting pathogenic bacteria that include species in the genera *Borrelia*, *Rickettsia*, *Francisella*, *Ehrlichia*, *Anaplasma*, and *Coxiella*, ticks also harbor bacterial endosymbionts which can have commensal, mutualistic, or parasitic relationships with their tick hosts [10-12]. The study of bacterial communities in ticks that transmit disease-causing agents has revealed new microbial associations including previously unknown tick-borne pathogens or vector competencies [13-15]. Elucidating the taxonomic composition of symbiotic bacteria facilitates our understanding of phylogenetic relationships between symbionts and the evolutionary biology of their association with tick hosts [16]. Microbial interactions within the tick host may influence pathogen characteristics and dynamics including transmission [17,18]. Additionally, the functional and genomic characterization of endosymbionts could provide opportunities for genetic engineering whereby transformants could be developed for use as microbial acaricides.

Molecular methods offer an expedient and efficient opportunity to analyze bacterial communities in ticks avoiding the need for intensive culture-based techniques, and furthermore, allow the identification of species which are not amenable to culturing. Specifically, pyrosequencing of partially amplified 16S rRNA sequences has been applied to study the composition of bacteria associated with biological systems including insect vectors [19-21]. Here, we evaluated bacterial diversity associated with *R. microplus *using bTEFAP. Bacterial composition was investigated in the egg, adult male and female life stages, and ovary and gut tissues from adult female cattle ticks. This report represents the first comprehensive survey of bacterial communities associated with the cattle tick using a culture-independent method.

## Results

### Estimated richness and diversity of bacterial communities

The application of bTEFAP reported here enabled us to explore the genome of bacterial symbionts, i.e. the microbiome, living inside and outside the cattle tick *R. microplus *as a means to initiate the characterization of the microbiota associated with this tick species of economic significance in animal agriculture worldwide. A total of 183,626 sequences were generated and a total of 130,019 sequences utilized for analyses of the 18 samples. Thus, an average of 7200 sequences > 350 bp (avg length 450 bp) per sample were analyzed after all quality control and screening steps. Indices of bacterial richness and diversity, based on Operational Taxonomic Unit (OTU) estimated through Rarefaction curve, Ace, and Chao1 procedures, are summarized in Table 1. Rarefaction and Richards maximum predicted curve modeling indicated that > 98% of OTUs at the 5% divergence were achieved for each sample [22], which suggests adequate depth of coverage (data not shown). Although results are presented at the 1, 3, and 5% dissimilarity levels, attention is focused on OTUs at 5% dissimilarity since it has been reported that reasonable genus-level richness can be achieved using that degree of discrimination [22]. By rarefaction analysis estimates, the trend for genera richness at 5% dissimilarity was: egg>gut > adult male > adult female > ovary.

**Table 1:** Estimated operational taxonomic units in samples of *Rhipicephalus (Boophilus) microplus* through Rarefaction, Ace, and Chao1.

Sample	Rarefaction*			Ace			Chao1		
	
	1%	3%	5%	1%	3%	5%	1%	3%	5%
Egg	576	388	361	780	466	433	696	427	396
Adult Male	299	128	98	452	167	124	457	174	125
Adult Female	237	110	93	339	143	117	366	154	138
Ovary	146	82	74	133	59	51	113	48	39
Gut	435	289	259	617	386	339	531	338	300

*Values are averaged for adult male and female (n = 2), and egg (n = 3) samples.

### Identification and quantification of bacterial taxa

**Figure 1:**
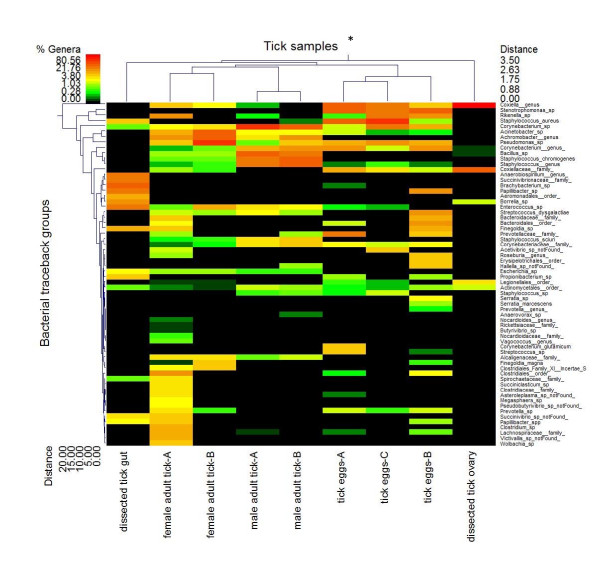
**Heat map depicting bacterial diversity and relative abundance in life stages and tissue samples from *R. microplus***. * Letters (A-C) used to identify individual life stage samples where applicable. Double hierarchical dendogram shows different bacteria distribution between taxonomic levels based on complete linkage clustering, and Manhattan distance methods with no scaling. Dendrogram linkages and distance of the bacterial taxa or traceback groups are not phylogenetic, but based upon relative abundance of the taxa within the samples. Traceback means bacterial classifications were based upon the percent identity of the sample sequence to known sequences, the percent divergence was then used to adjust identifications to the taxonomic level with the highest degree of confidence (e.g. a percent divergence < 3% can be expected to provide confidence at the species level, > 3% but < 5% at the genera level, etc.). Classifications were compiled after traceback. Legend and scale shown in upper left corner of the figure represent colors in heat map associated with the relative percentage of each traceback grouping of bacteria (cluster of variables in Y-axis) within each tick sample (X-axis clustering). Tick samples along the X-axis with Manhattan distances are indicated by branch length and associated with the scale located at the upper right corner of the figure. Bacterial traceback groups along the Y-axis are also clustered according to Manhattan distances; the respective scale is indicated in the figure's lower left corner.

In addition to surveying bacterial diversity across tick life stages and tissues, pyrosequencing also allowed assessment of the relative abundance of the taxonomic levels of bacteria detected (Figure 1). Tracebacks entered as "sp" in Figure 1 indicate that the characterization required for identification at the species level exists, but consensus on the particular nomenclature was lacking at the time groupings were done. However, tracebacks with the suffix "_genus" indicate that they may represent novel bacterial species. Genera that may include previously undescribed species of bacteria associated with the cattle tick include *Coxiella*, *Achromobacter*, *Corynebacterium*, *Staphyloccocus*, *Anaerobiospirillum*, *Roseburia*, *Prevotella*, *Nocardioides*, and *Vagoccocus*.

Bacteria identified to the species level include *Staphylococcus aureus*, *Staphylococcus chromogenes*, *Streptococcus dysgalactiae*, *Staphylococcus sciuri*, *Serratia marcescens*, *Corynebacterium glutamicum*, and *Finegoldia magna*. *Staphylococcus aureus* was present in adult males, eggs, and the gut of adult female cattle ticks. Similar findings were reported for the closely related tick species *Rhipicephalus decoloratus* and *Rhipicephalus geigyi* in Africa where *S. aureus* was isolated from the hemolymph of adult females and their eggs [23]. However, there was no evidence of transovarial transmission for *S. aureus* in those tick species. We detected *S. chromogenes* in adult male and female ticks. *Staphylococcus chromogenes* was isolated previously from *R. microplus* collected in Australia using a culture-dependent approach after the ticks had been surface-sterilized [24]. *Staphylococcus chromogenes* is part of the natural skin flora of cattle but can cause mastitis, and in pigs it may provoke exudative epidermitis [25,26].

The other five bacterial species represent previously unreported associations for *R. microplus*. Whereas *C. glutamicum *and *S. marcescens *were detected in eggs only, *S. sciuri *was present in male and female ticks, *F. magna *in eggs and female ticks, and *S. dysgalactiae *in eggs, male ticks, and female ticks. Because of our permissive approach to assess bacterial diversity, e.g., the ticks used in this study were not disinfected prior to DNA extraction, the prevalence of these new bacterial associations with *R. microplus *needs to be confirmed. However, it is relevant to note that *S. dysgalactiae *and *S. marcescens *are known to cause bovine mastitis, whereas *F. magna *was detected in papillomatous digital dermatitis lesions of cattle [27-29]. *Staphylococcus sciuris *is commonly found in the skin of cattle and other animals, while the natural habitats of *C. glutamicum *include soil, soils contaminated with bird feces, sewage and manure, and vegetables and fruits [30,31]. In their natural environment, *R. microplus *eggs may be exposed to *C. glutamicum *after oviposition by gravid female ticks.

Clustering analysis showed that the microbial biota detected in the ovary tissue of adult female ticks was the most dissimilar tissue of all the tick samples tested (Figure 1). Additionally, the least diverse microbial biota was detected in this tissue. Members of the Coxiellaceae family were the most prevalent bacteria in cattle tick ovary. Consistent with this finding, the Coxiellaceae were also found in the egg and adult female samples (Figure 1).

### Relative abundance of bacterial genera by tick life stage and tissue sample

One hundred twenty-one bacterial genera were detected in all the life stages and tissues sampled in this study (see Additional File 1 Table S1). Among the genera found in our study, *Arthrobacter*, *Bacillus*, *Curtobacterium*, *Enterobacter*, *Microbacterium*, *Paenibacillus*, *Pantoea*, *Pseudomonas*, *Rhodococcus*, *Serratia*, *Staphylococcus*, and *Stenotrophomonas *are genera previously reported to be harbored by *R. microplus *isolated from ticks collected in Australia [24]. *Enterobacter*, *Pseudomonas*, and *Staphylococcus*, found in both our study and the Australian study, were also cultured from homogenates of *R. microplus *in Bangladesh that were produced following surface sterilization and dissections using sterile technique [32]. Infection with *Achromobacter *and *Escherichia *was previously reported for cattle ticks from the Bangladesh study but not the Australian study.

Among the life stages sampled, the total number of bacterial genera detected in the egg, adult male, and adult female ticks was 54, 53, and 61, respectively (Additional File 1 Table S1). Of those numbers, 25, 25, and 27 genera were unique to the egg, adult male, and adult female life stages, respectively. By comparison, only 7 bacterial genera were identified in tick ovary, whereas 11 genera were found in the tick gut. *Cryobacterium*, *Rhodococcus*, and *Veillonella *were identified only in the ovary, whereas *Anaerobiospirillum *was the only genera unique to the gut.

**Figure 2:**
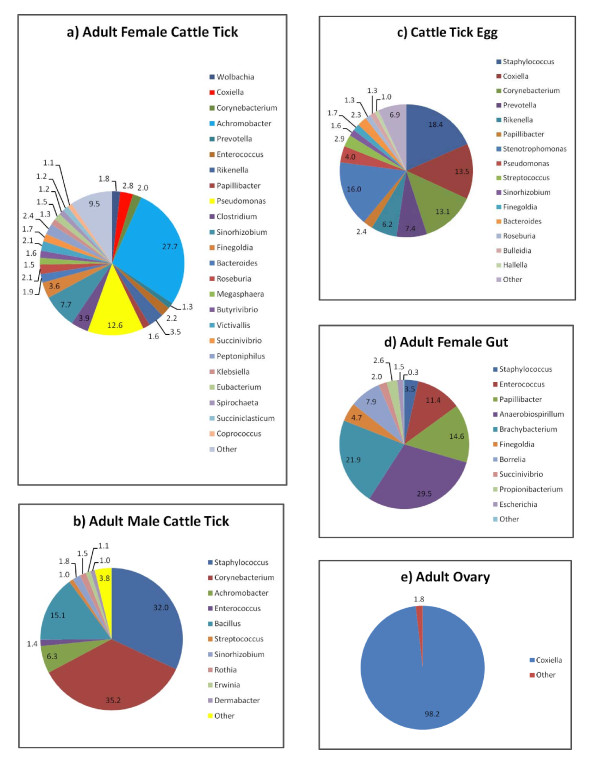
**Relative abundance of bacterial genera in life stages and tissue samples from *R. microplus *as detected by bTEFAP pyrosequencing**. a) Adult female cattle tick. Mean percentages (n = 2). Values below 1% were grouped as "Other" with total value of 9.5%. "Other" group includes: *Staphylococcus *(0.7%), *Bacillus *(0.5%), *Streptococcus *(0.7%), *Vagococcus *(0.3%), *Pseudobutyrivibrio *(0.7%), *Nocardioides *(0.2%), *Asteroleplasma *(0.9%), *Ruminococcus *(0.4%), *Escherichia *(0.9%), *Acetivibrio *(0.3%), *Erwinia *(0.1%), *Pedobacter *(0.2%), *Dermabacter *(0.1%), *Ornithinicoccus *(0.2%), *Oribacterium *(0.7%), *Alkaliflexus *(0.2%), *Paludibacter *(0.5%), *Pantoea *(0.2%), *Cytophaga *(0.1%), *Mitsuokella *(0.1%), *Enterobacter *(0.1%), *Paucisalibacillus *(0.4%), *Lachnobacterium *(0.1%), *Caldithrix *(0.2%), *Shigella *(0.1%), *Solirubrobacter *(0.1%), *Rhodobacter *(0.1%), *Desulfosporosinus *(0.1%). b) Adult male cattle tick. Mean percentages (n = 2). Values below 1% were grouped as "Other" with total value of 3.8%. "Other" group includes: *Coxiella *(0.1%), *Prevotella *(0.3%), *Rikenella *(0.1%), *Pseudomonas *(0.2%), *Escherichia *(0.3%), *Hallella *(0.3%), *Pantoea *(0.1%), *Moraxella *(0.7%), *Arthrobacter *(0.1%), *Enhydrobacter *(0.1%), *Mogibacterium *(0.1%), *Kocuria *(0.5%), *Enterobacter *(0.1%), *Exiguobacterium *(0.2%), *Lysinibacillus *(0.1%), *Belnapia *(0.1%). c) Cattle tick egg. Mean percentages (n = 3). Values below 1% were grouped as "Other" with total value of 6.9%. "Other" group includes: *Achromobacter *(0.3%), *Enterococcus *(0.1%), *Clostridium *(0.1%), *Serratia *(0.7%), *Ruminococcus *(0.3%), *Propionibacterium *(0.4%), *Klebsiella *(0.2%), *Acetivibrio *(0.9%), *Pedobacter *(0.6%), *Alkaliflexus *(0.4%), *Centipeda *(0.5%), *Pantoea *(0.1%), *Brevibacterium *(0.2%), *Rubrivivax *(0.4%), *Enhydrobacter *(0.2%), *Rhodoferax *(0.3%), *Sporocytophaga *(0.1%), *Alkanindiges *(0.2%), *Sphingopyxis *(0.1%), *Caulobacter *(0.1%), *Trichococcus *(0.1%), *Comamonas *(0.1%), *Anaerotruncus *(0.1%), *Akkermansia *(0.1%), *Legionella *(0.1%). d) Adult female cattle tick gut. Pool of tissue from five ticks tested. Values below 1% were grouped as "Other" with total value of 0.3%. "Other" group includes: *Corynebacterium *(0.3%). e) Adult cattle tick ovary. Pool of tissue from five ticks tested. Values below 1% were grouped as "Other" with total value of 1.8%. "Other" group includes: *Borrelia *(0.9%), *Cryobacterium *(0.9%).

The molecular approach applied in this study allowed us to assess the relative abundance of the microbiota associated with *R. microplus*. The predominant genera in the bacterial communities of the tick samples analyzed based on an abundance cutoff of 1.0% are shown for each sample in Figure 2. *Staphylococcus *was relatively abundant ( > 18%) in adult males and eggs, but not in adult female ticks. Other prevalent genera were *Corynebacterium *( > 13%) in eggs and adult males, and *Coxiella *( > 13%) in tick eggs. *Achromobacter *(27.7%), *Pseudomonas *(12.6%), and *Sinorhizobium *(7.7%) were the predominant genera found in adult female ticks. Among the tissues sampled, *Coxiella *was the most abundant (98.2%) genus in ovary, whereas *Anaerobiospirillum *(29.5%) and *Brachybacterium *(21.9%) predominated in the tick gut. Other relatively less abundant genera, but worth noting, include *Borrelia *(7.9%) in the tick gut; *Clostridium *(3.9%) in adult female ticks; *Escherichia *(1.5%) in the tick gut; *Klebsiella *(1.3%) in adult female ticks; *Streptococcus *in eggs (2.9%) and adult males (1.%); *Enterococcus *in adult male ticks (1.4%), adult female ticks (2.2%), and tick gut (11.4%); and *Wolbachia *in adult female ticks (1.8%).

## Discussion

To our knowledge, this study represents the first exploration of the diversity of the bacterial biota associated with distinct life stages and tissues of the cattle tick, *R. microplus *using a nonculturable method. Previous surveys of bacterial diversity in *R. microplus *employed culture methods, and for the most part, those studies focused on the isolation of bacteria pathogenic to the tick and vertebrate hosts [24,32-34]. The tag-encoded pyrosequencing approach reported here allowed us to detect and identify bacteria that otherwise might be fastidious, obligate intracellular, or noncultivable. Surveys of bacteria based on 16S rRNA gene sequences have proven useful to analyze the microbiome of bacterial communities in different habitats on and inside the host's body [35]. Our understanding of the ecology and eco-pathogenic relevance of tick-bacterial relationships is expanding as new associations are revealed through 16S rRNA gene-based analyses [14,36,37]. We probed deeply into the cattle tick microbiome using the 16S-bTEFAP technique. One hundred seven bacterial genera reported here represent new microbial associations for *R. microplus*. It has been suggested that the analysis of individual ticks could increase the ability to recognize bacteria in low copy numbers whereas the analysis of dissected organs would exclude the detection of external environmental bacteria [36]. We took a mixed approach by sampling ticks individually, without sterilization and prior to DNA isolation, for broad-range analysis of bacterial communities, while the gut and ovary were dissected for testing. Unique bacteria genera associations were detected for each of the tick samples tested. The symbiotic relationships for the bacterial genera associated with *R. microplus *remain to be characterized.

Although transovarial transmission enables bacterial colonization very early in the tick life cycle, copulation and egg fertilization could augment bacteria-tick associations through possibly infected sperm or the microbiota associated with the female genital tract [38]. It remains to be determined if antimicrobial activity occurs in *R. microplus *ejaculate, as has been shown for other arthropod species [39,40]. The environment where eggs are deposited influences the type of bacterial communities they are exposed to, which in some cases can include bacteria pathogenic to engorged females [41]. Ecological factors related to questing behavior facilitate contact with bacteria in the environment and expand the complexity of bacterial communities residing on a tick's exoskeleton. Further investigation of the microbiota in the tick exoskeleton is needed to understand the ecology of that microbial habitat in the context of host-microbe and microbe-microbe interactions. Studies in other biological systems have revealed the complexity of such interactions that offer the opportunity to develop novel diagnostic and therapeutic interventions [42,43], which in the context of this study could translate into options for tick biological control. Once on the host, ticks come in contact with the skin microbiota and become exposed to infected blood to fulfill their obligate hematophagous habit, or other host body fluids, while searching for and attaching at predilection sites. Systemic infection with bacteria acquired from the host skin, including *S. marcescens*, was documented in *Dermacentor andersoni *following a stringent, sterile sample processing protocol prior to tick trituration and media inoculation with the resulting suspension [44]. Here, it is documented that *R. microplus *harbors *S. marcescens*. Isolation of the bacterial genera *Staphylococcus *from *R. annulatus *and *R. decoloratus*, and *Streptococcus *from *R. annulatus *without specific characterization was reported previously [41,45,46]. Thus, systemic infection of *R. microplus *with *S. sciuri *and *S. dysgalactiae *may have occurred through host skin contact. This route of infection could also apply to *F. magna *because of its presence in the host skin habitat. Since *C. glutamicum *was detected in eggs laid by females collected in the field, it is possible that the ticks acquired the bacterium from hosts exposed to environmental sources. Given their economic impact on livestock production systems, our results indicate cattle transmission studies are warranted using *R. microplus *infected with *S. dysgalactiae*, *S. marcescens*, and *F. magna*.

The detection of *S. chromogenes *in cattle ticks from Australia and outbreaks in the USA, as well as the suite of bacterial genera shared by specimens from Australia, Bangladesh, and the USA noted here suggest that there may be a core microbiome associated with *R. microplus*. Alternatively, bacteria found in common between *R. microplus*, *R. annulatus*, *R. decoloratus*, and *R. geigyi *indicates that microbiota composition is influenced by the ecological niche they occupy during the parasitic stage, i.e. cattle. More extensive surveys are required to ascertain the biogeography of the microbiome across time and space as well as among and between *R. microplus *populations. As it has been shown for other anthropod vector-bacteria systems, these studies will help determine if bacterial communities associated with *R. microplus *represent random assemblages and define the influence of biotic and abiotic factors [14,21,37]. However, special attention is needed to harmonize sampling methods and molecular protocols given the rapid development of massively parallel sequencing technologies to facilitate meaningful comparisons. Additionally, it has been hypothesized that at least two tick species have evolved under the *R. microplus *designation [47]. The apparent co-evolution of certain bacterial lineages with their hosts warrants the application of that concept to test the hypothesis of genetic and reproductive divergence between geographic strains of *R. microplus *[12,47-49].

The *Coxiella*-type microbe we detected in *R. microplus *can be presumed to be an endosymbiont. Although more abundant in adult females, ovary, and eggs, a weak signal for the *Coxiella *microbe was noticed in one male tick. A similar observation was reported for a *Coxiella *endosymbiont in *Amblyomma americanum *[14,37]. Its presence in ovary and eggs indicates that the putative *R. microplus*-associated *Coxiella *endosymbiont can be transmitted vertically. Most of the bacterial sequences detected in the ovary were ascribed to the *Coxiella *microbe. This may result from selective amplification of the *Coxiella *symbiont associated with the expansion of ovarian tissue that takes place during engorgement since the ovary was collected from replete female *R. microplus *undergoing active oviposition [37,50]. The degree of relatedness between the *R. microplus*-associated *Coxiella *symbiont, *Coxiella *endosymbionts in other tick species, and *C. burnetii *remains to be determined. This will facilitate testing the hypothesis that the *R. microplus*-associated *Coxiella *microbe is a primary endosymbiont as documented for the *Coxiella *spp. infecting *A. americanum*, which showed a reduced genome in comparison to *C. burnetii *[50,51]. *Rhipicephalus microplus *has been found to harbor *C. burnetii *in India and China [52,53]. Our inability to detect *C. burnetii *in *R. microplus *from outbreaks in the USA suggests that the pathogen is not circulating in that tick population; alternatively, its presence in very low numbers prevented detection through the method used in this study. The concept of targeting endosymbionts as a means to control ticks and tick-borne diseases has been tested taking the chemotherapeutic approach [54,55]. Using antibiotics to treat the infection of *A. americanum *with a *Coxiella *spp. endosymbiont resulted in reduced reproductive fitness [55]. Novel approaches for endosymbiont isolation and characterization will facilitate *in vitro *culture to produce reagents for testing of the immunological approach to control ticks targeting their endosymbionts [54,56].

Our understanding of the associations between *R. microplus *and members of the genus *Borrelia *keeps expanding. *Borrelia theileri*, the etiologic agent of bovine borreliosis, has been shown to be transmitted by *R. microplus *in many parts of the world [57]. *Borrelia burgdorferi *was isolated from *R. microplus *in China [58]. Detection of the *R. microplus*-associated *Borrelia *in the gut and ovary reported here parallels the systemic infection with *B. theileri *where no adverse effects were observed in tick viability [33,59]. Like the *Borrelia *DNA sequences detected in this study, specific identification awaits for other *Borrelia *microbes isolated from *R. microplus *in diverse geographic locations [60-62]. However, *R. microplus *may be acting as a bridging vector facilitating the transmission of microbes across vertebrate hosts and possibly influencing ecological and evolutionary aspects of their natural history. The degree of similarity at the nucleotide level between a Mexican isolate of *B. theileri *and *Borrelia *spp. infecting *A. americanum *from the Northeast region of the USA suggests recent divergence [63]. Because white-tailed deer and cattle used to be sympatric throughout the southern USA prior to 1943, which is when cattle ticks were officially eradicated, it has been hypothesized that spirochetes infecting *A. americanum *may represent a host shift of *B. theileri *as *R. microplus *could have transmitted the spirochete to both ungulate hosts [64]. A *Borrelia *spp. detected in *R. microplus *from Brazil was shown to be closely related to *B. theileri *and *Borrelia lonestari *and the cattle tick-deer relationship was suggested as a natural process for the spread and/or maintenance of *Borrelia *spp. [65].

Although bacteria in the genus *Wolbachia *are generally found in reproductive tissues, the *R. microplus*-associated *Wolbachia *was not detected in ovarian tissue, but in the two adult female ticks assayed individually. Since ticks from a laboratory colony established in 1999 were the source of the ovarian tissue samples, it is plausible that *Wolbachia *infection was lost during the colonization process. It is also possible that laboratory rearing conditions allowed the *Coxiella *strain in the *R. microplus *ovaries sampled to out-compete pre-existing *Wolbachia *microbes with the eventual loss of infection in La Minita strain. Detection of the *Wolbachia-*type microbe in adult female ticks does not necessarily mean that the ovary was the only tissue infected. Disseminated *Wolbachia *infection has been documented in other arthropod vector species and similar events were reported for a *Coxiella *endosymbiont infecting *A. americanum *where the salivary glands were also infected [50,66]. The possibility for horizontal transmission would exist if *Wolbachia *infection of the *R. microplus *salivary glands were to occur. The horizontal transmission of *Wolbachia *microbes has been documented to occur more often than previously thought [67-69]. However, it has been shown in mosquitoes that the size of *Wolbachia *symbionts would prevent its free passage through the salivary ducts [70].

The functional relevance of our findings and observations needs to be tested. Proof of active infection is suggested to confirm the physiological significance of bacterial DNA detection in *R. microplus *by tag-encoded pyrosequencing or any other molecular or non-culturable approach. *Rhipicephalus microplus *has evolved various defense mechanisms acting in the hemocel if the external physical barrier represented primarily by the exoskeloton is bridged. Antimicrobial peptides form part of the cattle tick immune system [71,72]. Additionally, at least two types of *R. microplus *hemocytes exist that effect phagocytosis and production of reactive oxygen species [73]. Other components of the cattle tick immune system are likely to be discovered as additional functions are identified and assigned to the hemocyte transcriptome [74]. Caution must also be exercised in defining the relationship of bacteria found to be associated with *R. microplus *in this study. Although a particular genus may include pathogenic species, several of the bacterial genera detected and reported here for the first time in association with the cattle tick comprise groups commonly found in soil, on the surface of plants, or considered enteric bacteria. However, similar results from studies where stringent surface-sterilization was performed and negative controls were tested indicate that such bacteria are truly associated with *R. microplus *[14,37]. Lastly, blood feeding has been shown to increase bacterial diversity [37]. Thus, comparative analyses of the *R. microplus *microbiome between immature stages, unfed and blood-fed ticks across life stages, laboratory colony and wild-caught specimens, and additional organs and tissues are warranted [37].

It is worth noting that certain bacteria were detected in *R. microplus *by investigators in other parts of the world. *Rhipicephalus microplus *was found to harbor *Rickettsia conorii *in India [52]. *Ehrlichia canis *and a new *Ehrlichia *species closely related to *Ehrlichia chaffeensis *were detected in cattle ticks in China and the Thai-Myanmar border [53,58,75]. Additionally, *R. microplus *in the Caribbean contained *Ehrlichia ruminantium *DNA [76]. Our findings suggest that these pathogens of economic importance to livestock production systems are not circulating among outbreak strains of *R. microplus *in the USA. However, those studies highlight the potential role of *R. microplus *as vector of zoonotic bacteria. Although it is considered a rare event, *R. microplus *can parasitize humans [77,78].

The analysis of our results in the context of previous bacterial surveys provides an indication of geographic variation in the assemblages of bacteria associated with *R. microplus*. Additional reports on the identification of new bacterial species maintained in nature by *R. microplus *that may be pathogenic to its vertebrate hosts are expected as our understanding of its microbiota expands. Increased awareness of the role *R. microplus *can play in the transmission of pathogenic bacteria will enhance our ability to mitigate its economic impact on animal agriculture globally. This recognition should be included as part of analyses to assess the risk for re-invasion of areas where *R. microplus *was eradicated like the USA.

## Conclusion

Tick microbiomes remain largely unexplored. By comparison to the proposed strategy to accomplish the Human Microbiome Project, the work presented here constitutes the initial data acquisition and analysis exercise towards a comprehensive analysis of the *R. microplus *microbiome. A thorough understanding of the functional, ecological, and evolutionary aspects of the bacterial diversity in communities associated with the cattle tick requires additional investigations. The bacteria we found could have favorable effects on the tick's successful infestation of its cattle host, perhaps with roles in host blood digestion, immunity against infection by competing microbes potentially deleterious to the tick, or effects on population structure and fertility. Cattle ticks have evolved in conjunction with bovine hosts; therefore, bovine-tick interactions have likely influenced the ecology of their microbiomes. Even within the tick itself, there are feedback mechanisms influencing interactions at the host-microbiome interface. Our results further document the co-infection of cattle ticks with several bacteria, even in the presence of antimicrobial factors that are known to be produced by the tick immune system response in their hemolymph and gut tissues. Further investigations on the cattle tick microbiome are likely to enhance our understanding of the roles this cosmopolitan species serves as vector of bacteria that may be pathogenic to its vertebrate hosts.

## Methods

### Tick samples

Adult male and female ticks were obtained from a *R. microplus *infestation outbreak on cattle from Starr County, TX. Samples from the infestation were collected by USDA personnel in November, 2008, and shipped to the USDA Cattle Fever Tick Research Laboratory in Moore Field, TX, where the samples were frozen at -80°C. Prior to freezing, eggs were collected from gravid females, mixed together, and pooled and labeled as f1 generation. A portion of these f1 eggs were used to establish a laboratory colony to obtain adult ticks as described previously [79]. Two adult females and two adult males developed from these f1 eggs and three small clumps of approximately 100 f1 eggs each were used for the DNA extraction and pyrosequencing. The gut and ovary samples were obtained from the f20 generation of the La Minita strain of *R. microplus *that has been maintained *Babesia*-free at the University of Idaho Holm Research Center since 1999. The founding ticks for this strain were originally collected in Starr County, TX, in 1996. Using standard protocols approved by the University of Idaho Institutional Animal Care and Use Committee, La Minita larvae were placed on a stanchioned calf and replete females collected and dissected under sterile phosphate-buffered saline during the period of active oviposition. The ventral cuticle was excised; the ovaries and gut separately removed, rinsed in sterile saline, and held on ice until 5 ticks were dissected. The tissues were frozen at -80°C. A clump (~5 mm diameter) of the frozen material was broken off and used for pyrosequencing analysis. All samples used in this study were collected under open benchtop conditions. Neither surface sterilization nor sterile dissection techniques were employed during sample preparation steps prior to DNA extractions.

### Pyrosequencing and analysis

Bacterial tag-encoded FLX amplicon pyrosequencing (bTEFAP) was conducted as described previously [19,20]. Our approach was modified slightly to utilize the Titanium sequencing platform rather than FLX (Roche Applied Science, Indianapolis, IN) to take advantage of the longer average read lengths generated by the Titanium methodology. Additionally, we used a single 35 cycle PCR step with Qiagen HotStar Master Mix and addition of 0.5U of HotStar HiFidelity Polymerase in each reaction (Qiagen Inc., Valencia, CA). Finally, sequences used for analysis had average read length of ~450 bp with sequencing extending from the 27F 5' GAG TTT GAT CNT GGC TCA G 3' to 519r 5' GTN TTA CNG CGG CKG CTG 3' in relation to *E. coli *16S extending across V1 and into the V3 ribosomal region (Research and Testing Laboratory, Lubbock, TX). Amplicon sequencing was performed based upon the manufacturers protocols (Roche Applied Science, Indianapolis, IN) for Titanium sequencing on FLX-titanium platform. Raw data from bTEFAP was screened and trimmed based upon quality scores of Phred20 average and binned into individual sample collections. Sequence collections were then depleted of chimeras using B2C2 [80].

The resulting files were then depleted of short reads (< 350 bp) and bacterial species identified using BlastN comparison to a quality controlled and manually curated database derived from the NCBI. Data was compiled and relative percentages of each bacterial identification were determined for each individual sample. Data was also compiled at each individual taxonomic level according to the NCBI taxonomy criteria as described previously [19,20]. Rarefaction, Ace, and Chao 1 analyses to estimate mathematically predicted diversity and richness in tick samples was performed with DOTUR as described elsewhere [22,81].

## Authors' contributions

FDG and GAS conceived and designed the study; KGB and FDG prepared samples and acquired data for sequence analysis; SED performed sequence and bioinformatics analyses; RA and AAPL analyzed and interpreted the data, and drafted the article. All authors read and approved the final manuscript.

## Supplementary Material

Additional file 1 **Table S1 - Bacterial genera detected in *R*. (*B*.) *microplus***. Bacterial genera detected in *R*. (*B*.) *microplus *samples. Click here for file

## References

[B1] de la Fuente J Estrada-Peña A Venzal JM Kocan KM Sonenshine DE Overview: ticks as vectors of pathogens that cause disease in humans and animals Front Biosc 2008 13 6938 6946 10.2741/3200 18508706

[B2] Grisi L Massard CL Moya-Borja GE Pereira JB Impacto econômico das principais ectoparasitoses em bovinos no Brasil A Hora Veterinária 2002 21 8 10

[B3] Dutton JE Todd JL The nature of tick fever in the eastern part of the Congo Free State, with notes on the distribution and bionomics of the tick Br Med J 1905 2 1259 1260

[B4] Ricketts HT Some aspects of Rocky Mountain spotted fever as shown by recent investigations Med Rec 1909 76 843 855 10.1093/clinids/13.6.1227 1775857

[B5] Hotez PJ Kamath A Neglected tropical diseases in sub-Saharan Africa: review of their prevalence, distribution, and disease burden PloS Negl Trop Dis 2009 3 e412 10.1371/journal.pntd.0000412 19707588 PMC2727001

[B6] Heyman P Cochez C Hofhuis A van der Giessen J Sprong H Porter SR Losson B Saegerman C Donoso-Mantke O Niedrig M Papa A A clear and present danger: tick-borne diseases in Europe Expert Rev Anti Infect Ther 2010 8 33 50 10.1586/eri.09.118 20014900

[B7] Parola P Raoult D Ticks and tickborne bacterial diseases in humans: an emerging infectious threat Clin Inf Dis 2001 32 897 928 10.1086/319347 11247714

[B8] Schouls LM Van De Pol I Rijpkema SG Schot CS Detection and identification of *Ehrlichia*, *Borrelia burgdorferi *sensu lato, and *Bartonella *species in Dutch *Ixodes ricinus *ticks J Clin Microbiol 1999 37 2215 2222 10364588 10.1128/jcm.37.7.2215-2222.1999 PMC85121

[B9] Cowdry EV A group of microorganisms transmitted hereditarily in ticks and apparently unassociated with disease J Exp Med 1925 41 817 830 10.1084/jem.41.6.817 19869029 PMC2130977

[B10] Noda H Munderloh UG Kurtti TJ Endosymbionts of ticks and their relationship to *Wolbachia spp*. and tick-borne pathogens of humans and animals Appl Environ Microbiol 1997 63 3926 3932 9327557 10.1128/aem.63.10.3926-3932.1997 PMC168704

[B11] Sacchi L Bigliardi E Corona S Beninati T Lo N Franceschi A A symbiont of the tick *Ixodes ricinus *invades and consumes mitochondria in a mode similar to that of the parasitic bacterium *Bdellovibrio bacteriovorus* Tissue Cell 2004 36 43 53 10.1016/j.tice.2003.08.004 14729452

[B12] Scoles GA Phylogenetic analysis of the *Francisella*-like endosymbionts of *Dermacentor *ticks J Med Entomol 2004 41 277 286 10.1603/0022-2585-41.3.277 15185926

[B13] Burgdorfer W Brinton LP Hughes LE Isolation and characterization of symbionts from the Rocky Mountain wood tick, *Dermacentor andersoni* J Invert Pathol 1973 22 424 434 10.1016/0022-2011(73)90173-0 4202564

[B14] Clay K Klyachko O Grindle N Civitello D Oleske D Fuqua C Microbial communities and interactions in the lone star tick, *Amblyomma americanum* Mol Ecol 2008 17 4371 4381 10.1111/j.1365-294X.2008.03914.x 19378409

[B15] Vilcins IE Fournier P Old JM Deane E Evidence for the presence of *Francisella *and spotted fever group Rickettsia DNA in the tick *Amblyomma fimbriatum *(Acari: Ixodidae), Northern territory, Australia J Med Entomol 2009 46 926 933 10.1603/033.046.0427 19645299

[B16] Rymaszewska A Symbiotic bacteria in oocyte and ovarian cell mitochondria of the tick *Ixodes ricinus*: biology and phylogenetic position Parasitol Res 2007 100 917 920 10.1007/s00436-006-0412-8 17226040

[B17] Macaluso KR Sonenshine DE Ceraul SM Azad AF Rickettsial infection in *Dermacentor variabilis *(Acari: Ixodidae) inhibits transovarial transmission of a second Rickettsia J Med Entomol 2002 39 809 813 10.1603/0022-2585-39.6.809 12495176

[B18] de la Fuente J Blouin EF Kocan KM Infection exclusion of the rickettsial pathogen *Anaplasma marginale *in the tick vector *Dermacentor variabilis* Clin Diagn Lab Immun 2003 10 182 184 10.1128/CDLI.10.1.182-184.2003 PMC145288 12522060

[B19] Dowd SE Sun Y Wolcott RD Domingo A Carroll JA Bacterial tag-encoded FLX amplicon pyrosequencing (bTEFAP) for microbiome studies: bacterial diversity in the ileum of newly weaned *Salmonella*-infected pigs Foodborne Pathog Dis 2008 5 459 472 10.1089/fpd.2008.0107 18713063

[B20] Dowd SE Callaway TR Wolcott RD Sun Y McKeehan T Hagevoort RG Edrington TS Evaluation of the bacterial diversity in the feces of cattle using 16S rDNA bacterial tag-encoded FLX amplicon pyrosequencing (bTEFAP) BMC Microbiol 2008 8 125 10.1186/1471-2180-8-125 18652685 PMC2515157

[B21] Jones TR Knight R Martin AP Bacterial communities of disease vectors sampled across time, space, and species ISME J 2010 4 223 231 10.1038/ismej.2009.111 19865184

[B22] Acosta-Martínez V Dowd S Sun Y Allen V Tag-encoded pyrosequencing analysis of bacterial diversity in a single soil type as affected by management and land use Soil Biol Chem 2008 40 2762 2770

[B23] Amoo AO Dipeolu OO Akinboade AO Adeyemi A Bacterial isolation from and transmission by *Boophilus decoloratus *and *Boophilus geigyi* Folia Parasitol 1987 34 69 74 3108116

[B24] Murrel A Dobson SJ Yang X Lacey E Barker SC A survey of bacterial diversity in ticks, lice and fleas from Australia Parasitol Res 2003 89 326 334 12632173 10.1007/s00436-002-0722-4

[B25] Devriese LA Baele M Vaneechoutte M Martel A Haesebrouk F Identification and antimicrobial susceptibility of *Staphylococcus chromogenes *isolates from intramammary infections of dairy cows Vet Microbiol 2002 87 175 182 10.1016/S0378-1135(02)00047-0 12034545

[B26] Andresen LO Ahrens P Daugaard L Bille-Hansen V Exudative epidermitis in pigs caused by toxigenic *Staphylococcus chromogenes* Vet Microbiol 2005 105 291 300 10.1016/j.vetmic.2004.12.006 15708827

[B27] Garvie EI Farrow JAE Bramley AJ *Streptococcus dysgalactiae *(Diernhofer) nom. rev Int J Syst Bacteriol 1983 33 404 405 10.1099/00207713-33-2-404

[B28] Bannerman DD Paape MJ Goff JP Kimura K Lippolis JD Hope JC Innate immune response to intramammary infection with *Serratia marcescens *and *Strepococcus uberis* Vet Res 2004 35 681 700 10.1051/vetres:2004040 15535958

[B29] Yano T Moe KK Yamazaki K Ooka T Hayashi T Misawa N Identification of candidate pathogens of papillomatous digital dermatitis in dairy cattle from quantitative 16S rRNA clonal analysis Vet Microbiol 2010 143 352 362 10.1016/j.vetmic.2009.12.009 20036086

[B30] Nagase N Sasaki A Yamashita K Shimizu A Wakita Y Kitai S Kawano J Isolation and species distribution of staphylococci from animal and human skin J Vet Med Sci 2002 64 245 250 10.1292/jvms.64.245 11999444

[B31] Liebl W Eggeling L, Bott M From *Corynebacterium *Taxonomy Handbook of Corynebacterium glutamican 2005 Florida: Taylor & Francis Group 9 36 full_text

[B32] Rahman MH Rahman MM Occurrence of some bacterial isolates in ticks found in Madhupur Forest Area Bang Vet Jour 1980 14 43 47

[B33] Smith RD Brener J Osorno M Ristic M Pathobiology of *Borrelia theileri *in the tropical cattle tick, *Boophilus microplus* J Invertebr Pathol 1978 32 182 190 10.1016/0022-2011(78)90028-9 731072

[B34] Brum JGW Teixeira MO Acaricidal activity of *Cedecea lapagei *on engorged females of *Boophilus microplus *exposed to the environment Arq Bras Med Vet Zoot 1992 44 543 544

[B35] Turnbaugh PJ Ley RE Hamady M Fraser-Liggett CM Knight R Gordon JI The human microbiome project Nature 2007 449 804 810 10.1038/nature06244 17943116 PMC3709439

[B36] Schabereiter-Gurtner C Lubitz W Rölleke S Application of broad-range 16S rRNA PCR amplification and DGGE fingerprinting for detection of tick-infecting bacteria J Microbiol Meth 2003 52 251 260 10.1016/S0167-7012(02)00186-0 12459246

[B37] Heise SR Elshahed MS Little SE Bacterial diversity in *Amblyomma americanum *(Acari: Ixodidae) with a focus on members of the genus *Rickettsia* J Med Entomol 2010 47 258 268 10.1603/ME09197 20380308

[B38] Afzelius BA Alberti G Dallai R Godula J Witalinski W Virus- and Rickettsia-infected sperm cells in arthropods J Invertebr Path 1989 53 365 377 10.1016/0022-2011(89)90102-X

[B39] Joseph L Josekumar VS George PV Detection of antimicrobial activity in accessory gland secretions of the virgin male red palm weevil, *Rhynchophorus ferrugineus* Internet J Microbiol 2009 7 1

[B40] Otti O Naylor RA Siva-Jothy MT Reinhardt K Bacteriolytic activity in the ejaculate of an insect Am Nat 2009 174 292 295 10.1086/600099 19548839

[B41] Hendry DA Rechav Y Acaricidal bacterial infecting laboratory colonies of the tick *Boophilus decoloratus *(Acarina: Ixodidae) J Invertebr Pathol 1981 38 149 151 10.1016/0022-2011(81)90044-6 7024424

[B42] Fierer N Lauber CL Zhou N McDonald D Costello EK Knight R Forensic identification using skin bacterial communities PNAS 2010 107 6477 6481 10.1073/pnas.1000162107 20231444 PMC2852011

[B43] Iwase T Uehara Y Shinji H Tajima A Seo H Takada K Agata T Mizunoe Y *Staphylococcus epidermidis *Esp inhibits *Staphylococcus aureus *biofilm formation and nasal colonization Nature 2010 456 346 349 10.1038/nature09074 20485435

[B44] Steinhaus EA The microbial flora of the Rocky Mountain Wood Tick, *Dermacentor andersoni *Stiles J Bacteriol 1942 44 397 404 16560577 10.1128/jb.44.4.397-404.1942 PMC373689

[B45] Ahmed LS Dosoky RM Some bacterial isolates from *Boophilus annulatus *ticks under natural conditions in Assiut Governorate Assuit Vet Med J 1986 15 199 202

[B46] El Kammah KM Oyoun LMI Abdel-Shafy S Detection of microogranisms in the saliva and midgut smears of different tick species (Acari: Ixodoidea) in Egypt J Egypt Soc Parasitol 2007 37 533 539 17985586

[B47] Labruna MB Naranjo V Mangold AJ Thompson C Estrada-Pena A Guglielmone AA Jongejan F de la Fuente J Allopatric speciation in ticks: gentic and reproductive divergence between geographic strains of *Rhipicephalus (Boophilus) microplus* BMC Evol Biol 2009 9 46 10.1186/1471-2148-9-46 19243585 PMC2656471

[B48] Hongoh Y Deevong P Inoue T Moriya S Trakulnaleamsai S Ohkuma M Vongkaluang C Noparatnaraporn N Kudo T Intra- and interspecific comparsions of bacterial diversity and community structure support coevolution of gut microbiota and termite host Appl Environ Microb 2005 71 6590 6599 10.1128/AEM.71.11.6590-6599.2005 PMC1287746 16269686

[B49] Dethlefsen L McFall-Ngai M Relman DA An ecological and evolutionary perspective on human-microbe mutualism and disease Nature 2007 449 811 818 10.1038/nature06245 17943117 PMC9464033

[B50] Klyachko O Stein BD Grindle N Clay K Fuqua C Localization and visualization of a *Coxiella*-type symbiont within the lone star tick, *Amblyomma americanum* Appl Environ Microb 2007 73 6584 6594 10.1128/AEM.00537-07 PMC2075054 17720830

[B51] Jasinskas A Zhong J Barbour AG Highly prevalent *Coxiella *sp. bacterium in the tick vector *Amblyomma americanum* Appl Environ Microb 73 334 336 10.1128/AEM.02009-06 PMC1797106 17085709

[B52] Padbidri VS Rodrigues JJ Shetty PS Joshi MV Rao BL Shukla RN Tick-borne rickettsioses in Pune district, Maharashtra, India Int J Zoonoses 1984 11 45 52 6500861

[B53] Wen B Cao W Pan H *Ehrlichiae *and ehrlichial diseases in China Ann NY Acad Sci 2003 990 45 53 10.1111/j.1749-6632.2003.tb07335.x 12860598

[B54] Ghosh S Azhahianambi P Yadav MP Upcoming and future strategies of tick control: a review J Vect Borne Dis 2007 44 79 89 17722860

[B55] Zhong J Jasinskas A Barbour AG Antibotic treatment of the tick vector *Amblyomma americanum *reduced reproductive fitness PLoS ONE 2007 2 e405 10.1371/journal.pone.0000405 17476327 PMC1852332

[B56] Mediannikov O Sekeyová Z Birg ML Raoult D A novel obligate intracellular gamma-proteobacterium associated with Ixodid ticks, *Diplorickettsia massiliensis*, gen. nov., sp. nov PLoS ONE 2010 5 e11478 10.1371/journal.pone.0011478 20644718 PMC2903611

[B57] Matton P Van Melckebeke H Bovine borreliosis: comparison of simple methods for detection of the spirochaete in the blood Trop Anim Hlth Prod 1990 22 147 152 10.1007/BF02241005 2219454

[B58] Wen B Jian R Zhang Y Chen R Simultaneous detection of *Anaplasma marginale *and a new *Ehrlichia *species closely related to *Ehrlichia chaffeensis *by sequences analyses of 16S ribosomal DNA in *Boophilus microplus *ticks from Tibet J Clin Microbiol 2002 40 3286 3290 10.1128/JCM.40.9.3286-3290.2002 12202567 PMC130830

[B59] Smith RD Miranpuri GS Adams JH Ahrens EH *Borrelia theileri*: isolation from ticks (*Boophilus microplus*) and tick-borne transmission between splenectomized calves Am J Vet Res 1985 46 1396 1398 4026019

[B60] Callow LL Hoyte HMD Transmission experiments using *Babesia bigemina*, *Theileria mutans*, *Borrelia *sp. and the cattle tick, *Boophilus microplus* Aust Vet J 1961 73 381 390 10.1111/j.1751-0813.1961.tb03790.x

[B61] Rodríguez Vivas RI Cen Aguilar F Domínguez Alpízar JL Cob Galera LA Solís Calderon JJ Detección de espiroquetas del género *Borrelia *en hemolinfas de teleoginas de *Boophilus microplus *en el estado de Yucatán, México Vet Mex 1996 27 187 188

[B62] Rezende J Kessler RH Soares CO Martins OP Ocorrência de *Borrelia *spp. em cultura de células embrionárias do carrapato *Boophilus microplus *(Acari: Ixodidae) no estado do Mato Grosso do Sul, Brasil Rev Brasileira Parasitol Veter 2008 17 50 52 10.1590/s1984-29612008000100011 18554442

[B63] Rich SM Armstrong PM Smith RD Telford SR III Lone star tick-infecting Borrelia are most closely related to the agent of bovine borreliosis J Clin Microbiol 2001 39 494 497 10.1128/JCM.39.2.494-497.2001 11158095 PMC87764

[B64] Spielman A Pollack RJ Telford SR III Ginsberg HS The origins and course of the present outbreak of Lyme disease Ecology and environmental management of Lyme Disease 1992 New Jersey: Rutgers University Press 83 96

[B65] Yparraguirre LA Machado-Ferreira E Ullmann AJ Piesman J Zeidner NS Soares CAG A hard tick relapsing fever group spirochete in a Brazilian *Rhipicephalus (Boophilus) microplus* Vector-Borne Zoonot Dis 2007 7 717 721 10.1089/vbz.2007.0144 17979536

[B66] Moreira LA Iturbe-Ormaetxe I Jeffery JA Lu G Pyke AT Hedges LM Rocha BC Hall-Mendelin S Day A Riegler M Hugo LE Johnson KN Kay BH McGraw EA van den Hurk AF Ryan PA O'Neill SL A *Wolbachia *symbiont in *Aedes aegypti *limits infection with Dengue, Chikungunya, and *Plasmodium* Cell 2009 139 1268 1278 10.1016/j.cell.2009.11.042 20064373

[B67] Vavre F Fleury F Lepetit D Fouillet P Bouletreau M Phylogenetic evidence for horizontal transmission of *Wolbachia *in host-parasitoid associations Mol Biol Evol 1999 16 1711 1723 10605113 10.1093/oxfordjournals.molbev.a026084

[B68] Ahrens ME Shoemaker D Evolutionary history of *Wolbachia *infections in the fire ant *Solenopsis invicta* BMC Evol Biol 2005 5 35 10.1186/1471-2148-5-35 15927071 PMC1175846

[B69] Viljakainen L Reuter M Pamilo P *Wolbachia *tranmission dynamics in *Formica *wood ants BMC Evol Biol 2008 8 55 10.1186/1471-2148-8-55 18291041 PMC2277377

[B70] Moreira LA Saig E Turley AP Ribeiro JMC O'Neil SL McGraw EA Human probing behavior of *Aedes aegypti *when infected with a life-shortening strain of *Wolbachia* PLoS Negl Trop Dis 2009 3 e568 10.1371/journal.pntd.0000568 20016848 PMC2788697

[B71] Fogaça AC Lorenzini DM Kaku LM Esteves E Bulet P Daffre S Cysteine-rich antimicrobial peptides of the cattle tick *Boophilus microplus*: isolation, structural characterization and tissue expression profile Dev Comp Immunol 2004 28 191 200 14642886 10.1016/j.dci.2003.08.001

[B72] Fogaça AC Almeidae IC Eberlin MN Tanaka AS Bulet P Daffre S Ixodidin, a novel antimicrobial peptide from the hemocytes of the cattle tick *Boophilus microplus *with inhibitory activity against serine proteinases Peptides 2006 27 667 674 16191451 10.1016/j.peptides.2005.07.013

[B73] Pereira LS Oliveira PL Barja-Fidalgo C Daffre S Production of reactive oxygen species by hemocytes from the cattle tick *Boophilus microplus* Exp Parasitol 2001 99 66 72 10.1006/expr.2001.4657 11748959

[B74] Santos IK Valenzuela JG Ribeiro JM de Castro M Costa JN Costa AM da Silva ER Neto OB Rocha C Daffre S Ferreira BR da Silva JS Szabó MP Bechara GH Gene discovery in *Boophilus microplus*, the cattle tick Ann NY Acad Sci 2006 1026 242 246 10.1196/annals.1307.037 15604500

[B75] Parola P Cornet JP Sanogo YO Miller RS Van Thien H Gonzalez JP Raoult D Telford SR III Wongsrichanalai C Detection of *Ehrlichia *spp., *Anaplasma *spp., *Rickettsia *spp., and other eubacteria in ticks from the Thai-Mynmar border and Vietnam J Clin Microbiol 2003 41 1600 1608 10.1128/JCM.41.4.1600-1608.2003 12682151 PMC153861

[B76] Robinson JB Eremeeva ME Olson PE Thornton SA Medina MJ Sumner JW Dasch GA New approaches to detection and identification of *Rickettsia africae *and *Ehrlichia ruminatium *in *Amblyomma variegatum *(Acari: Ixodidae) Ticks From the Caribbean J Med Entomol 2009 46 942 951 10.1603/033.046.0429 19645301

[B77] Estrada-Peña A Jongejan F Ticks feeding on humans: a review of records on human-biting Ixodoidea with special reference to pathogen transmission Exp Appl Acarol 1999 23 685 715 10581710 10.1023/a:1006241108739

[B78] Girotto A Zangirolando A Teixeira Y Vidotto O de Moraes GJ, Castilho RC, Flechtmann Parasitism by *Rhipicephalus (Boophilus) microplus *(Canestrini, 1887) in humans in the northern part of Parana State, Brazil13th International Congress of Acarology Abstract Book: 23-27 August 2010; Brazil 2010 92 93

[B79] Miller RJ Li AY Tijerina M Davey RB George JE Differential response to diazinon and coumaphos in a strain of *Boophilus microplus *(Acari: Ixodidae) collected in Mexico J Med Entomol 2008 45 905 911 10.1603/0022-2585(2008)45[905:DRTDAC]2.0.CO;2 18826034

[B80] Gontcharova V Youn E Wolcott RD Hollister EB Gentry TJ Dowd SE Black box chimera check (B2C2): a windows-based software for batch depletion of chimeras from bacterial 16S rRNA gene datasets Open Microbiol J 2010 4 47 52 10.2174/1874285801004010047 PMC3040993 21339894

[B81] Schloss PD Handlesman J Introducing DOTUR, a computer program for defining operational taxonomic units and estimating species richness Appl Environ Microbiol 2005 71 1501 1506 10.1128/AEM.71.3.1501-1506.2005 15746353 PMC1065144

