# Supplementary material for: Assessment of bacterial diversity in the cattle tick Rhipicephalus (Boophilus) microplus through tag-encoded pyrosequencing
Source: BMC Microbiol. 2011 Jan 6;11:6. doi: 10.1186/1471-2180-11-6 (PMC3025832; doi:10.1186/1471-2180-11-6)
Supplement: Additional file 1 — Table S1 - Bacterial genera detected in R. (B.) microplus. Bacterial genera detected in R. (B.) microplus samples. [file 1471-2180-11-6-S1.PDF]

Table S1 – Bacterial genera detected in *R. (B.) microplus*.

Bacterial genus	Detection in *R.* (*B.*) *microplus**
Acetivibrio	AF, EG
Achromobacter	AF, AM, EG
Acidimicrobium	AF
Aeromonas	AF
Akkermansia	EG
Alkaliflexus	AF, EG
Alkanindiges	EG
Anaerobiospirillum	GT
Anaerostipes	EG
Anaerotruncus	EG
Anaerovorax	AM
Arcanobacterium	AM
Arcobacter	EG
Arthrobacter	AM
Asteroleplasma	AF, EG
Bacillus	AF, AM, OV
Bacteroides	AF, EG
Belnapia	AM
Borrelia	GT, OV
Brachybacterium	AM, EG, GT
Brevibacterium	EG
Brevundimonas	EG
Bulleidia	EG
Butyrivibrio	AF, AM
Caldithrix	AF
Caryophanon	AM
Caulobacter	EG
Cellulomonas	AM
Cellvibrio	EG
Centipeda	EG
Clostridium	AF, AM, EG
Comamonas	EG
Conexibacter	AM
Coprococcus	AF, EG

Corynebacterium	AF, AM, EG, GT, OV
Coxiella	AF, AM, EG, OV
Cryobacterium	OV
Cupriavidus	EG
Curtobacterium	AM
Cystobacter	AM
Cytophaga	AF
Dermabacter	AF, AM
Desulfosporosinus	AF
Dorea	EG
Enhydrobacter	AM, EG
Enterobacter	AF, AM, EG
Enterococcus	AF, AM, EG, GT
Erwinia	AF, AM
Escherichia	AF, AM, GT
Eubacterium	AF
Exiguobacterium	AM
Finegoldia	AF, EG, GT
Geobacillus	AF
Hallella	AM, EG
Herbaspirillum	AM
Jeotgalicoccus	AM
Klebsiella	AF, EG
Kocuria	AM
Lachnobacterium	AF, AM
Lachnospira	AF
Legionella	EG
Leptothrix	EG
Lysinibacillus	AF, AM
Macrococcus	AM
Massilia	AM
Megasphaera	AF
Methylibium	AF
Microbacterium	AM
Micrococcus	AM
Mitsuokella	AF
Mogibacterium	AM
Moraxella	AM

Mycobacterium	AF
Neisseria	AM, EG
Nocardioides	AF
Oribacterium	AF
Ornithinicoccus	AF
Paenibacillus	AM
Paludibacter	AF
Pantoea	AF, AM, EG
Papillibacter	AF, AM, EG, GT
Paracoccus	AM
Paucisalibacillus	AF
Pedobacter	AF, EG
Peptoniphilus	AF
Phenylobacterium	AM
Prevotella	AF, AM, EG
Propionibacterium	EG, GT
Pseudobutyrivibrio	AF
Pseudomonas	AF, AM, EG
Rhodobacter	AF, AM
Rhodococcus	OV
Rhodoferax	EG
Rikenella	AF, AM, EG
Roseburia	AF, AM, EG
Rothia	AM
Rubrivivax	EG
Ruminococcus	AF, EG
Selenomonas	AF
Serratia	EG
Shigella	AF, AM
Sinorhizobium	AF, AM, EG
Solirubrobacter	AF
Sphingomonas	AM
Sphingopyxis	EG
Spirochaeta	AF
Sporobacterium	AF
Sporocytophaga	EG
Staphylococcus	AF, AM, EG, GT
Stenotrophomonas	EG
Streptococcus	AF, AM, EG

Succiniclasticum	AF
Succinivibrio	AF, GT
Thauera	EG
Thermomicrobium	AM
Trichococcus	EG
Turicibacter	EG
Vagococcus	AF
Veillonella	OV
Victivallis	AF
Wolbachia	AF

*** AF - Adult Female Tick; AM - Adult Male Tick; EG - Egg; GT - Adult Female Gut; OV - Ovary**
